# The Impact of Supervisor Support on the Job Satisfaction of Immigrant and Minority Long-Term Care Workers

**DOI:** 10.1093/geroni/igab046.3647

**Published:** 2021-12-17

**Authors:** Frances Hawes, Shuangshuang Wang

**Affiliations:** 1 University of Wisconsin-Eau Claire, Eau claire, Wisconsin, United States; 2 Shandong University, School of Public Health, Cheeloo College of Medicine, Jinan, Shandong, Shandong, China (People's Republic)

## Abstract

The need for long-term care workers (LTCW) will grow significantly as the American population ages. Understanding the factors that impact job satisfaction of this workforce has important implications for policy and practice. Previous research has demonstrated the effect of supervisor support on the job satisfaction of these workers; however, much less is known about how this effect differs among different race/ethnicity or immigration groups. This study examined how supervisor support mediates the associations between race/ethnicity, immigration status, and job satisfaction among nursing assistants (NAs). Data of 2,763 NAs were extracted from the National Nursing Assistant Survey (2004). Race/ethnicity groups included White (54%), African American (30%), Asian (2%), Hispanic (10%), and others (4%). Immigration status included U.S.-born citizens (87%), naturalized (7%) and resident/alien (6%). Bivariate analyses showed that Asian NAs perceived higher levels of supervisory support than other races, whereas U.S.-born NAs reported lower levels of supervisory support than naturalized and residents/aliens. Findings from multivariate analyses indicated that non-Hispanic Asians and Resident/Alien workers reported significantly higher levels of job satisfaction than their counterparts, and the associations were fully mediated by NAs’ perceived supervisor support. These findings support prior research that supervisor support is important to improving job satisfaction and contribute to the literature that Asians/Residents/Aliens long-term care workers may be more sensitive to supervisory support and may be more grateful if they received support from supervisors. Managers should be aware of these racial differences and by being supportive they may improve NAs job satisfaction and reduce turnover rates.

